# Dihydrotanshinone I Inhibits the Lung Metastasis of Breast Cancer by Suppressing Neutrophil Extracellular Traps Formation

**DOI:** 10.3390/ijms232315180

**Published:** 2022-12-02

**Authors:** Huan Zhao, Yi Liang, Chengtao Sun, Yufei Zhai, Xuan Li, Mi Jiang, Ruiwen Yang, Xiaojuan Li, Qijin Shu, Guoyin Kai, Bing Han

**Affiliations:** 1 Laboratory for Core Technology of TCM Quality Improvement and Transformation, School of Pharmaceutical Science, The Third Affiliated Hospital, Academy of Chinese Medical Science, Zhejiang Chinese Medical University, Binwen Road 548, Binjiang District, Hangzhou 310053, China; 2 Department of Oncology, The First Affiliated Hospital, Zhejiang Chinese Medical University, Hangzhou 310053, China

**Keywords:** breast cancer, lung metastasis, dihydrotanshinone I, neutrophil extracellular traps, RNA sequencing, *TIMP1*

## Abstract

Breast cancer (BC) is a common female malignancy, worldwide. BC death is predominantly caused by lung metastasis. According to previous studies, Dihydrotanshinone I (DHT), a bioactive compound in *Salvia miltiorrhiza* Bunge (*S. miltiorrhiza*), has inhibitory effects on numerous cancers. Here, we investigated the anti-metastatic effect of DHT on BC, where DHT more strongly inhibited the growth of BC cells (MDA-MB-231, 4T1, MCF-7, and SKBR-3) than breast epithelial cells (MCF-10a). Additionally, DHT repressed the wound healing, invasion, and migration activities of 4T1 cells. In the 4T1 spontaneous metastasis model, DHT (20 mg/kg) blocked metastasis progression and distribution in the lung tissue by 74.9%. DHT reversed the formation of neutrophil extracellular traps (NETs) induced by phorbol 12-myristate 13-acetate, as well as ameliorated NETs-induced metastasis. Furthermore, it inhibited Ly6G^+^Mpo^+^ neutrophils infiltration and H3Cit expression in the lung tissues. RNA sequencing, western blot, and bioinformatical analysis indicated that *TIMP1* could modulate DHT acting on lung metastasis inhibition. The study demonstrated a novel suppression mechanism of DHT on NETs formation to inhibit BC metastasis.

## 1. Introduction

Breast cancer (BC) is, at present, the most common cancer worldwide [1]. Triple negative breast cancer (TNBC) is characterized by the lack of an estrogen receptor, a progesterone receptor, and a human epidermal growth factor receptor 2 [2]. Although anthracycline and taxane-based drugs can improve life quality and survival time in the early stages, no effective treatment is available for TNBC patients [3,4]. High invasion and metastasis render treatment of TNBC difficult and primarily cause TNBC-patient death [5]. The TNBC cells have different organ metastasis tendencies (commonly lung, bone, liver, brain), among which, lung metastasis shows an only 16.8% of 5 year overall survival rate [6]. Although combined therapies can inhibit breast cancer metastasis, e.g., trastuzumab + paclitaxel (first-line drug) for metastatic BC, the side effects are irreversible [3]. Therefore, it is urgent to develop low-side-effect drugs that can prevent TNBC metastasis.

Neutrophils, the most abundant circulating leukocytes, are vital components of innate and adaptive immunity [7,8]. During infection, neutrophils migrate from the periphery to the tissue [9]. Neutrophils guard against pathogens through phagocytosis, the production of reactive oxygen species (ROS), the formation of neutrophil extracellular traps (NETs), etc., contributing to the host‘s defense system [10,11,12,13]. NETs, consisting of chromatin DNA scaffolds coated with granular proteins, promote BC metastasis by enhancing tumor cell migration to distant organs, including lung tissue [13,14,15]. Moreover, the content of NETs is positively associated with the lung metastasis of BC [16]. Thus, the inhibition of NETs-formation is a potential target for BC treatment.

*Salvia miltiorrhiza* Bunge (*S. miltiorrhiza*) is a well-known medicinal plant of the family Lamiaceae [17], and is widely used in cardio- and cerebro-vascular diseases [18]. The bioactive capacities of *S. miltiorrhiza* include anti-tumor, antioxidant, anti-bacterial, anti-inflammation, behavior [19,20]. Dihydrotanshinone I (DHT), a major compound in *S. miltiorrhiza*, has potent anti-tumor activity against breast, lung, liver, prostate, and ovarian cancer [21]. DHT was reported to inhibit the proliferation of BC cells MCF-7, MDA-MB-231, and BC stem cells [22,23]. However, its effect on lung metastasis of BC has not been thoroughly disclosed.

In this study, we investigated the anti-tumor activity of DHT on BC in vitro and in vivo. Our results showed that DHT effectively inhibited the cell viability, migration, and invasion of BC. We also confirmed its inhibitory ability on tumor growth and lung metastasis in 4T1 xenograft nude mice, which are mouse mammary carcinoma cells and are commonly used as a spontaneous metastasis model. Furthermore, DHT was demonstrated to decrease neutrophil infiltration, subsequently preventing NETs formation by controlling *TIMP1* (tissue inhibitor of matrix metalloproteinase-1, *TIMP1*) expression. Our study indicates the potential effect of DHT against BC, and provides a novel strategy for BC treatment.

## 2. Results

### 2.1. Tanshinones Inhibited Proliferation and Clonogenicity of BC Cells

**Figure 1:**
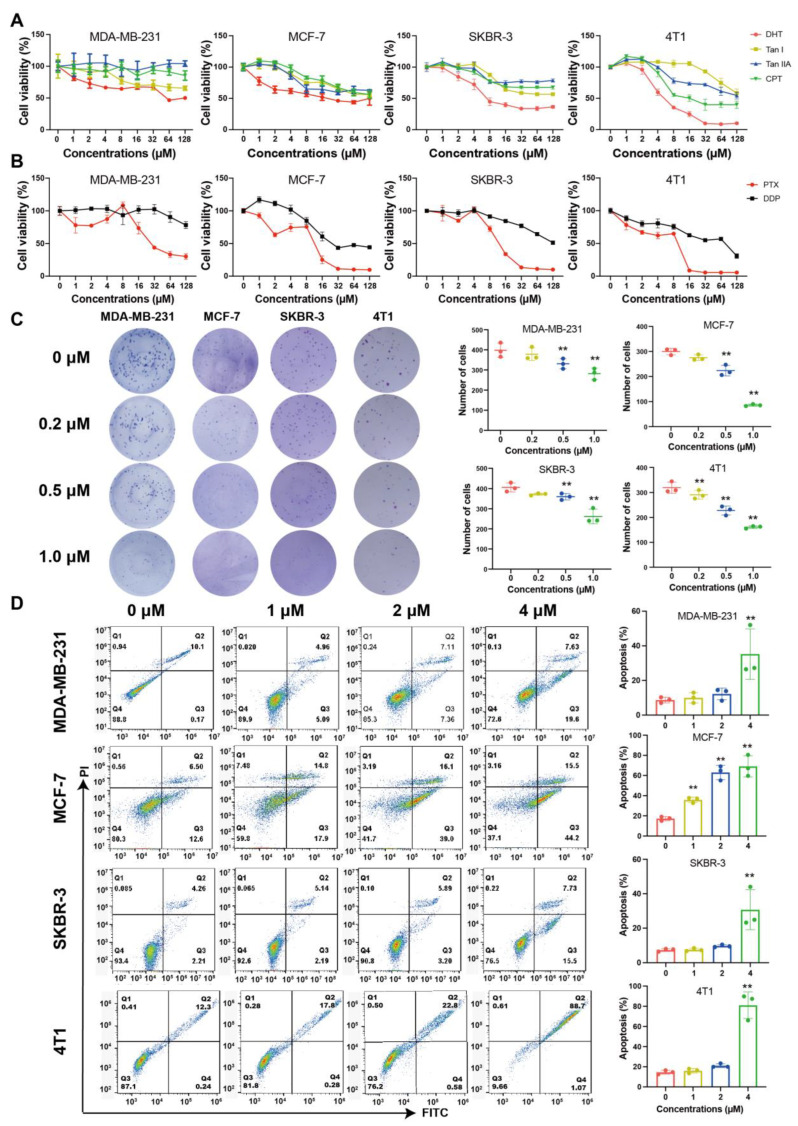
The effect of tanshinones on proliferation and clonogenicity of BC cells. MDA-MB-231, MCF-7, SKBR-3, and 4T1 cells were treated with four tanshinones, DDP, or PTX (1–128 μM) for 24 h, respectively. (**A**,**B**) Cell viability was determined by MTT assay. (**C**) MDA-MB-231, MCF-7, SKBR-3, and 4T1 cells were treated with DHT (0.2–1 μM) for 12 d. Clonogenicity was determined by crystal violet staining. (**D**) MDA-MB-231, MCF-7, SKBR-3, and 4T1 cells were treated with DHT (1–4 μM) for 24 h. Apoptosis was detected by flow cytometry using the Annexin V-FITC detection kit. Data were represented as mean ± SD, n = 3. ** *p* < 0.01 compared with the control group.

The effects of four tanshinones (DHT; tanshinone I, Tan I; tanshinone IIA, Tan IIA; cryptotanshinone, CPT) on different types of BC cells were evaluated. DHT showed the most significant inhibitory effect on the MDA-MB-231, MCF-7, SKBR3, and 4T1 cells. The IC_50_ value of 4T1 cells was the lowest (Figure 1A). Compared with the first-line clinical drugs, DHT showed a better inhibition rate than cisplatin (DDP) and had similar efficacy to paclitaxel (PTX) on four types of BC cells (IC_50_ of 4T1: 6.97 μM (DHT), 51.53 μM (DDP), 5.08 μM (PTX)) (Figure 1B, Table S1). Meanwhile, the inhibition rate of DHT on normal breast epithelial cells, MCF-10a, was lower than that of DDP and PTX (Figure S1). DHT significantly inhibited the clonogenicity of BC cells in the colony formation assay (Figure 1C). PTX and DDP almost entirely inhibited the clonogenicity of BC cells (Figure S2). The low concentration of DHT had a limited inhibitory effect on the BC cells. However, when the concentration of DHT reached 4 μM, the apoptosis rates in the MDA-MB-231, MCF-7, SKBR-3, and 4T1 cells were increased to 35.25%, 69.10%, 30.70%, and 81.04%, respectively (Figure 1D).

### 2.2. DHT Inhibited the Migration and Invasion of 4T1 Cells

**Figure 2:**
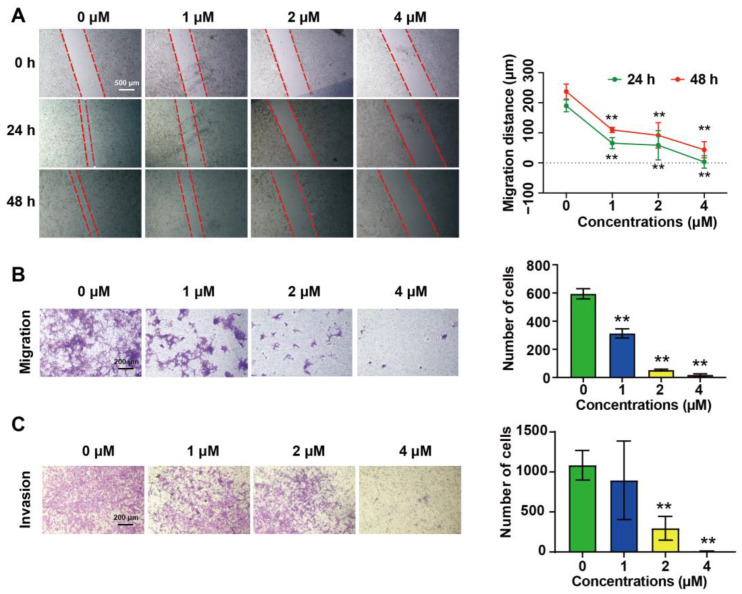
The effects of DHT on migration and invasion of 4T1 cells. (**A**) 4T1 cells were treated with DHT (1–4 μM) for 24 h or 48 h. The migration distance was detected by microscopes. (**B,C**) 4T1 cells were treated with DHT (1–4 μM) for 24 h, then collected and seeded in a transwell chamber with or without matrigel for 24 h (migration) or 48 h (invasion). The number of cells was detected by crystal violet staining. Data were represented as mean ± SD, n = 4. ** *p* < 0.01 compared with the control group.

DHT (1 μM, 2 μM, and 4 μM) inhibited the wound healing of the 4T1 cells in a dose-dependent manner (Figure 2A). The transwell assay showed that DHT (2 μM and 4 μM) significantly inhibited the migration (Figure 2B) and invasion (Figure 2C) of the 4T1 cells. Compared with the control group, the inhibition degree of DHT on the invasion ability was 72.7% (2 μM) and 99.1% (4 μM).

### 2.3. Induction of NETs and Its Correlation with BC

**Figure 3:**
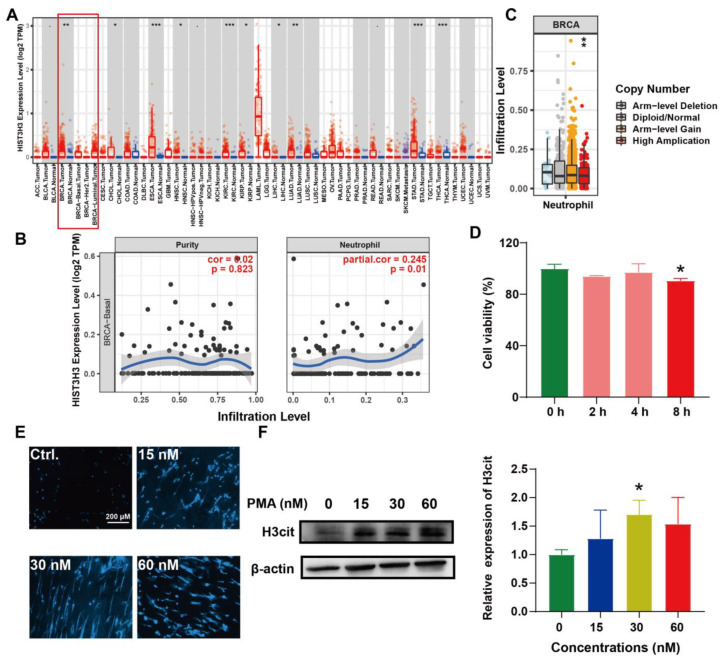
Induction of NETs and its association with BC. (**A**) The mRNA expression of *HIST3H3* in different cancers and adjacent normal tissues was analyzed by TIMER. Red box indicated the breast cancer and the adjacent normal tissues. (**B**) The correlation between *HIST3H3* expression and neutrophil infiltration in BC was analyzed by TIMER. (**C**) The correlation between copy number alteration of *HIST3H3* and neutrophil infiltration was analyzed by TIMER. (**D**) PMNs were extracted from the femur bone of ICR mice and cultured for 2 h, 4 h, and 8 h. Thereafter, the cell viability was determined by MTT assay. Data were represented as mean ± SD, n = 3. * *p* < 0.05 and ** *p* < 0.01 compared with the group of 0 h. (**E**) PMNs were treated with PMA (15–60 nM) for 6 h. The cell morphology was determined by Hoechst 33342 staining. (**F**) PMNs were treated with PMA (15–60 nM) for 6 h. The H3cit expression was determined by Western Blot. Data were represented as mean ± SD, n = 3. * *p* < 0.05, ** *p* < 0.01, and *** *p* < 0.001 compared with the control group.

High amounts of tumor-infiltrating neutrophils are associated with the adverse outcomes of BC patients. The pro-tumor phenotype of neutrophils is in part influenced by their ability to produce NETs [24]. Therefore, we performed bioinformatics analysis of the relationship among NETs, neutrophils, and BC. TIMER is a comprehensive database for the systematical analysis of immune infiltrates across diverse cancer types. The findings of this database revealed that *HIST3H3* (protein: H3cit, a biomarker of NETs) was significantly expressed in BC compared with adjacent normal tissues (Figure 3A). The *HIST3H3* level was positively correlated with polymorph nuclear neutrophils (PMNs) infiltration (Figure 3B). Meanwhile, the copy number alteration of *HIST3H3* significantly correlated with the PMNs infiltration levels (Figure 3C). It suggested that NETs were associated with BC development and PMNs infiltration. Thereafter, PMNs were extracted and cultured in vitro [11]. We found that the PMNs died significantly after 8 h incubation (Figure 3D). Meanwhile, phorbol 12-myristate 13-acetate (PMA), a NETs activator, induced morphological changes and increased the H3cit expression in PMNs (Figure 3E,F). These data indicated the successful establishment of the PMA-induced NETs formation model.

### 2.4. DHT Reversed NETs-Induced Proliferation, Migration, and ROS Production of 4T1 Cells

**Figure 4:**
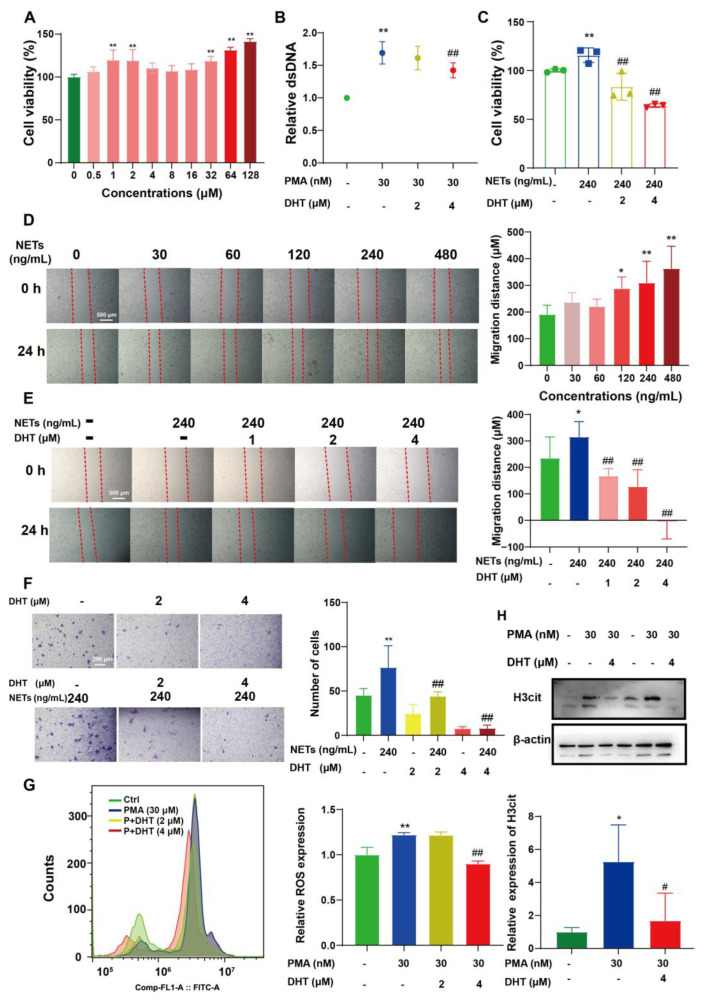
DHT reversed the proliferation, migration, and ROS production of 4T1 cells induced by NETs or PMA. (**A**) PMNs were treated with DHT (0.5–128 μM) for 8 h. The cell viability was detected by MTT assay, n = 3. (**B**) PMA-induced (30 nM) PMNs were treated with or without DHT (2 μM and 4 μM) for 8 h. The supernatant was collected and determined by microplate system, n = 3. (**C**) The NETs-treated 4T1 cells were treated with or without DHT (2 μM and 4 μM) for 24 h. The cell viability was detected by MTT assay, n = 3. (**D**) The 4T1 cells were treated with NETs (30–480 ng/mL) for 24 h. The migration distance was detected by a microscope, n = 4. (**E**) The NETs-treated (240 ng/mL) 4T1 cells were treated with DHT (1–4 μM) for 24 h. The migration distance was detected, n = 4. (**F**) The 4T1 cells were treated with or without NETs (240 ng/mL) and DHT (2 μM and 4 μM) for 24 h. The number of cells was detected by crystal violet staining, n = 3. (**G**) The PMA-induced (30 nM) PMNs were treated with DHT (2 μM and 4 μM) for 8 h. Intracellular ROS level was determined by flow cytometry using DCFH-DA probe, n = 3. (**H**) The PMA-induced (30 nM) PMNs were treated with DHT (4 μM) for 8 h. The H3cit expression was determined by Western Blot assay, n = 3. Data were represented as mean ± SD. * *p* < 0.05 and ** *p* < 0.01 compared with the control group. ^#^ *p* < 0.05 and ^##^ *p* < 0.01 compared with the PMA or NETs-treated groups.

Surprisingly, DHT significantly increased the cell viability of PMNs (Figure 4A). It blocked PMA-induced morphological changes (Figure S3) and the formation of NETs in PMNs (Figure 4B). In addition, NETs promoted the proliferation (Figure 4C) and migration (Figure 4D–F) of 4T1 cells, while DHT reversed these phenomena. ROS is a necessary factor for the formation of NETs, but DHT treatment blocked PMA-induced ROS generation (Figure 4G). In addition, DHT decreased PMA-induced H3cit expression in PMNs (Figure 4H).

### 2.5. DHT Suppressed the NETs Formation by Restraining TIMP1 Expression

**Figure 5:**
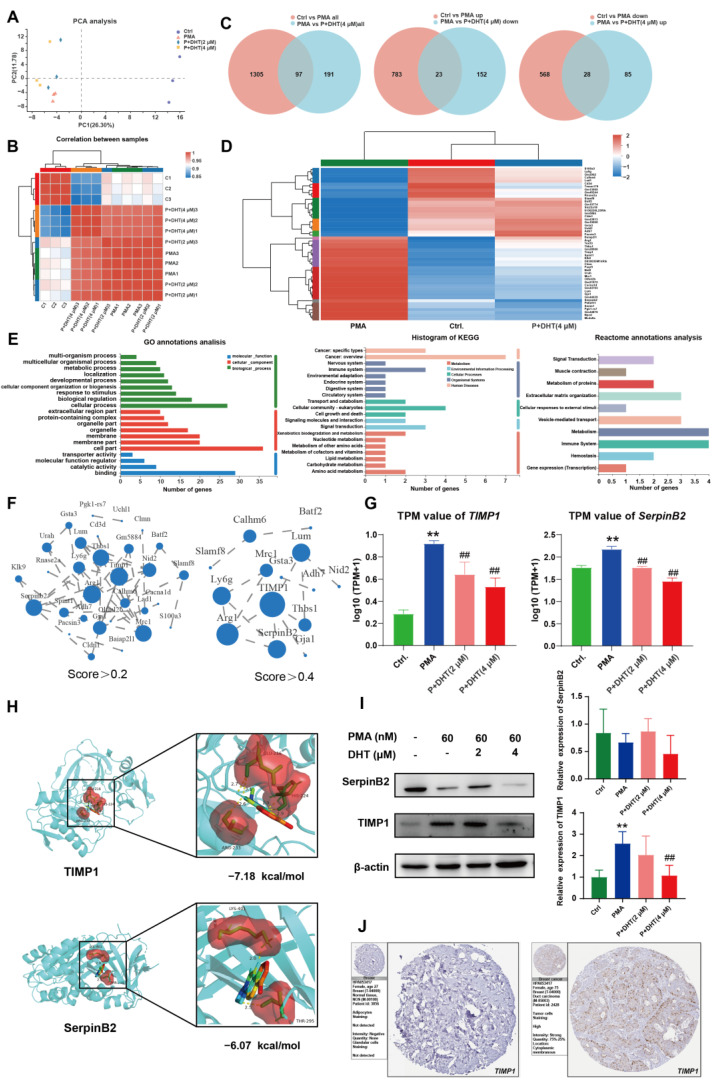
DHT suppressed NETs formation by restraining *TIMP1* expression. The PMA-induced (30 nM) PMNs were treated with DHT (2 μM and 4 μM) for 8 h. The total cDNA was sequenced using the Illumina HiSeq™ 300 platform. (**A**) PCA analysis. (**B**) Plots of correlation between samples in control, PMA (30 nM), P (30 nM) + DHT (2 μM), P (30 nM) + DHT (4 μM) groups. (**C**) The differentially expressed genes in control vs. PMA and PMA vs. P + DHT (4 μM) group with a cutoff of |log2fold change| ≥ 2. The differentially expressed genes were used for (**D**) heatmap analysis, (**E**) GO, KEGG, and Reactome annotation analysis, and (**F**) protein-protein interaction analysis. (**G**) The expression of *TIMP1* and *SerpinB2* in the control, PMA, P + DHT (2 μM), and P + DHT (4 μM) groups. (**H**) The molecule docking diagram of DHT with *TIMP1* and *SerpinB2* proteins. (**I**) The expression of *TIMP1* and *SerpinB2* in the PMA-induced PMNs was determined by Western Blot assay. (**J**) Immunohistochemical analysis of *TIMP1* from the breast of normal population and BC patients detected in the HPA database. Data were represented as mean ± SD, n = 3. ** *p* < 0.01 compared with the control group. ^##^ *p* < 0.01 compared with the PMA-treated group.

RNA sequencing was performed to further study the mechanism of DHT on the formation of NETs. PCA showed that the samples were well dispersed among each group (Figure 5A), with good within-correlation (Figure 5B). There were 97 common genes between control vs. PMA and PMA vs. P + DHT (4 μM). Specifically, 23 common genes were observed in the control vs. PMA (up group) and the PMA vs. P + DHT (down group), 28 common genes were observed in the control vs. PMA (down group) and the PMA vs. P + DHT (up group) (Figure 5C). The above 51 genes were defined as differentially expressed genes (Table S1). A heatmap showed that DHT could reverse the changes in gene expression caused by PMA (Figure 5D). GO, KEGG and Reactome analysis indicated that DHT mainly participated in cancer and the immune system (Figure 5E). The PPI protein interaction was analyzed with cutoff scores greater than 0.2 or 0.4, respectively. The data showed that *SerpinB2* and *TIMP1* genes played a crucial role (Figure 5F, Tables S2 and S3). Moreover, DHT significantly reversed PMA-induced *SerpinB2* and *TIMP1* expression (Figure 5G). Molecular docking indicated that DHT interacted with GLU-216, HIS-224 and ARG-233 residues in *TIMP1* (−7.18 kcal/mol), LYS-401 and THR-295 residues in *SerpinB2* protein (−6.07 kcal/mol) (Figure 5H). Western blotting confirmed that DHT significantly inhibited *TIMP1* expression in PMA-induced PMNs, but had no marked effect on *SerpinB2* expression (Figure 5I). The PHA database showed that high levels of *TIMP1* powerfully heralds poor clinical results for BC patients (Figure 5J). Together, *TIMP1* might be the target gene for the potential inhibition of DHT on the formation of NETs.

### 2.6. DHT Inhibited Tumor Growth and Lung Metastasis in 4T1 Tumor-Bearing Nude Mice

**Figure 6:**
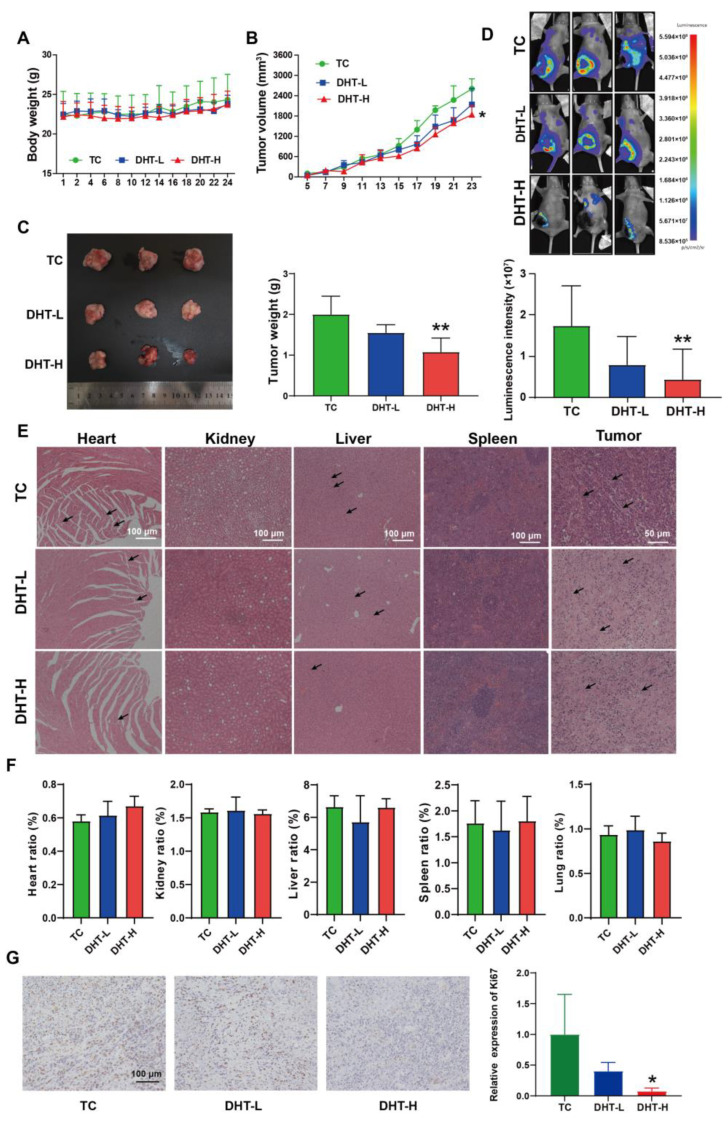
DHT suppressed tumor growth and lung metastasis in 4T1 tumor-bearing nude mice. The 4T1 cells (10^6^ cells/mice) were harvested, resuspended in RPMI-1640 medium, then implanted into the left breast pad of nude mice. The mice in DHT groups were daily intraperitoneally injected with 10 mg/kg (DHT-L) or 20 mg/kg (DHT-H). The mice in the TC group were intraperitoneally injected with saline. (**A**) Body weight. (**B**) Tumor volume. (**C**) Tumor weight. Data were represented as mean ± SD, n = 4. * *p* < 0.05 and ** *p* < 0.01 compared with the TC group. (**D**) The bioluminescence imaging of representative mouse and the luminescence intensity of the lungs. Data were represented as mean ± SD, n = 3. * *p* < 0.05 and ** *p* < 0.01 compared with the TC group. (**E**) H&E staining of the heart, kidney, liver, spleen, and tumor. Arrows indicated the pathological changes in the H&E section. (**F**) The organ ratios of the heart, kidney, liver, lung, and spleen were calculated at the end of the experiment. Data were represented as mean ± SD, n = 4. (**G**) Immunohistochemistry of Ki67 in the tumor tissues. Data were represented as mean ± SD, n = 3. * *p* < 0.05 compared with the TC group.

The 4T1 orthotopic nude mice model was established to evaluate the anti-tumor effect of DHT in vivo. After 25 days of treatment, DHT showed no effect on the body weight (Figure 6A), but significantly inhibited the tumor volume (Figure 6B) and tumor weight (Figure 6C), as well as the proliferation marker Ki67 level in the tumor tissues (Figure 6G). In vivo imaging showed that the lung fluorescence intensity was decreased by 74.9% in the DHT-H group compared with the TC group (Figure 6D), suggesting that DHT had an inhibitory effect on lung metastasis. H&E staining showed that DHT improved myocardial injury, decreased inflammatory cell infiltration in the liver, and induced tumor cell necrosis and nucleus fragmentation in the tumor tissue. The arrangement of the renal tubules was tighter and the red pulp boundary of the spleen became clearer after DHT treatment (Figure 6E). Meanwhile, no significant differences were observed in the organ ratios of heart, liver, spleen, lung, and kidney among the groups (Figure 6F).

**Figure 7:**
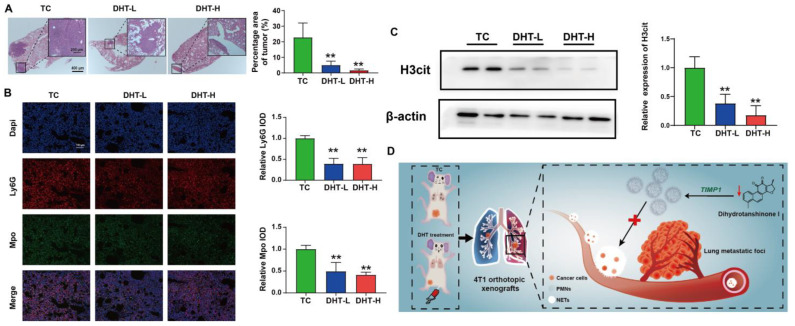
DHT suppressed neutrophils and H3cit expression in the lung tissues. (**A**) The H&E staining of lung tissue. (**B**) Immunofluorescence of neutrophils biomarker Ly6G and Mpo in the lung tissues. (**C**) The expression of H3Cit in the lung tissues was determined by Western Blot assay. (**D**) The mechanism diagram of DHT inhibiting BC lung metastasis. Data were represented as mean ± SD, n = 3. ** *p* < 0.01 compared with the TC group.

DHT decreased the area of metastatic foci in the lung tissue (Figure 7A). Compared with the TC group, DHT significantly reduced the expression of Ly6G and Mpo (neutrophils biomarker) in the lung tissues (Figure 7B). Similarly, DHT inhibited H3cit expression in the lung tissues (Figure 7C). These data suggest that DHT inhibited the number of neutrophils and the formation of NETs in the lung tissue of 4T1 xenograft nude mice.

## 3. Discussion

BC is one of the most common cancers and has high incidence and mortality rates [1]. TNBC, the most aggressive subtype, is easy to metastasize and has strong drug resistance [25]. Cisplatin is widely used in advanced BC treatment. However, approximately 50% of patients will rapidly develop acquired resistance [26]. Hence, it is urgent to find effective compounds with less toxicity and drug resistance. DHT, a main component in *S. miltiorrhiza*, has a significant inhibitory effect on various cancers including BC [27]. In this study, we found DHT significantly inhibited the proliferation of BC cells (MDA-MB-231, 4T1, MCF-7, and SKBR-3) with IC_50_ (117.71, 6.97, 34.11, 17.87 μM) lower than DDP (2613.12, 51.53, 50.90, 134.93 μM). Moreover, DHT suppressed the migration and invasion of 4T1 cells. In addition, the tumor growth and lung metastasis in nude mice were restrained after DHT administration. These results suggest that DHT could be a potential treatment for metastatic BC.

Neutrophils are the most abundant innate immune cells in the bone marrow and peripheral blood, and participate in immunity and inflammation progression. Recently, neutrophils were demonstrated to have deleterious effects in promoting cancer cell growth and metastasis [28]. NETs are neutrophils products and contain a group of components including Mpo, proteases, and histones. The formation of NETs in the tumor microenvironment is known to drive BC metastasis progression [29]. Moreover, the degree of BC lung metastasis is related to the NETs content [16]. Targeting NETs formation regulators was reported to prevent breast-to-lung metastasis, further decreasing lung metastatic niches [30,31]. In this study, we found that DHT could inhibit the death of PMNs in vitro. In addition, DHT reduced the H3cit expression in PMA-induced PMNs and the lung tissues of 4T1 xenograft nude mice. Similarly, the Ly6G expression in the lung tissues was also inhibited, which indicates the decrease in neutrophils infiltration. ROS plays a catalytic role in NETs formation [32]. Consistently, DHT treatment decreased ROS production. Therefore, it suggests that DHT might inhibit BC lung metastasis by suppressing NETs formation.

*TIMP1* is a glycoprotein, binding with matrix metalloproteinase to inhibit its proteolytic property [33]. *TIMP1* is also a biomarker of prognosis and chemotherapy response. The overexpression of *TIMP1* promotes cancer cell invasion and angiogenesis during BC development [34]. *TIMP1* directly triggers the formation of NETs in primary human neutrophils and is correlated with DNA-bound myeloperoxidase, which is a NETs marker. Furthermore, NETs predominantly colocalized in areas with increased *TIMP1* expression in patient-derived tumors [28]. Activated *TIMP1* promotes the expression of matrix-derived factor-1 in hepatic stellate cells and induces neutrophil migration, a marker of the premetastatic niche [35]. Here, we observed that DHT reversed PMA-induced *TIMP1* expression, which was substantially expressed in BC patients. KEGG analysis showed that DHT had effects on both the immune system and cancer. Indeed, *TIMP1* was previously demonstrated to correlate with immune markers such as M1 macrophage, M2 macrophage, tumor-associated macrophage, Tregs, and neutrophils [36]. This suggested that *TIMP1* may be a potential target of DHT in regulating NETs formation. *SerpinB2* is a paralog of *PAI1* and is overexpressed in the TNBC. A high level of *SerpinB2* was related to decreased survival and increased lung metastasis in BC patients [37]. However, DHT suppressed the *SerpinB2* mRNA level but had no appreciable impact on its protein expression. Further study is required to unveil the mechanism. As PMNs begin to die after 8 h incubation in vitro, the gene-editing and further validation in PMNs are challenging in this study. The results indicated that DHT blocked the NETs formation and BC lung metastasis, at least partially, by inhibiting *TIMP1* expression.

## 4. Materials and Methods

### 4.1. Reagents

DHT (purity ≥ 98.0%), Tan I (purity ≥ 98.0%), CT (purity ≥ 98.0%), Tan IIA (purity ≥ 98.0%), and D-luciferin potassium salt (purity ≥ 98.0%) were purchased from Yuanye Biotechnology (Shanghai, China). PMA, DDP, PTX, Dulbecco′s modified Eagle′s Medium (DMEM), RPMI-1640, 0.25% trypsin-EDTA, fetal bovine serum (FBS), and penicillin/streptomycin solution were purchased from Gibco (Grand Island 14072, NY, USA). Antibodies against β-actin, and goat anti-rabbit were purchased from Proteintech (Wuhan, China). Histone H3 (citrulline R2 + R8 + R17), Mpo, and Ly6G were purchased from Abcam (Cambridge, UK). Matrigel was purchased from Thermo Fisher Scientific (Waltham 02454, MA, USA). The dsDNA HS Assay Kit for Qubit was purchased from Yeasen (Shanghai, China). Crystal violet and ROS assay kits were purchased from Solarbio (Beijing, China).

### 4.2. Cell Culture

MDA-MB-231, MCF-7, SKBR-3, and 4T1 cells were bought from the Cell Bank of the Chinese Academy of Sciences (Shanghai, China). The MCF-10a cells were a gift from professor Huajun Zhao, Zhejiang Chinese medical university. The PMNs were extracted from ICR mice (6-weeks old), Shanghai Slack Laboratory Animal Co., Ltd. (SCXK (HU) 2017-0005). The cells were cultured at 37 °C with 5% CO_2_ in DMEM or RPMI-1640 medium containing 10% (*v/v*) fetal FBS and 1% (*v/v*) penicillin/streptomycin. MCF-10a cells were cultured with MEGM Mammary Epithelial Cell Growth Medium Bullet Kit (Lonza, Basel, Switzerland).

### 4.3. MTT Assay

The cytotoxicity was evaluated using the MTT assay (Solarbio, Beijing, China). The cells in the logarithmic growth phase were seeded in a 96-well plate (10^4^ cells/well). After incubation overnight, the cells were treated with DHT, CPT, Tan IIA, Tan I, PTX and DDP in medium containing 1% FBS for 24 h. Then, 10 μL of MTT solution (5 mg/mL, dissolved in PBS) was added to each well for 4 h. Thereafter, the medium was removed and 100 μL of DMSO was added to each well [38]. The absorbance of each well was measured at 490 nm by a microplate reader (BioTek Cytation 1, Winooski 05404, VT, USA).

### 4.4. Colony Formation Assay

The cells in the logarithmic growth phase were seeded in a 6-well plate (10^3^ cells/well) and treated with DHT, CPT, Tan IIA, and Tan I in a medium containing 10% FBS for 12 d. Thereafter, the cells were rinsed with PBS, then fixed with paraformaldehyde. The number of clones was counted after crystal violet staining.

### 4.5. Wound Healing and Transwell Assays

The metastasis ability was evaluated by the wound healing, migration, and invasion assays, according to previous methods [39]. The cells in the logarithmic growth phase were seeded in a 6-well plate (2 × 10^5^ cells/well). When the cells were full, a scratch was made in each well and the width of scratches was measured. Then, the cells were treated with DHT in RPMI-1640 medium containing 1% FBS for 24 h and 48 h. After treatment, the width of scratches was measured and captured under a microscope (Optec BDS400, Chongqing, China). As for the migration and invasion assays, 4T1 cells were harvested after DHT intervention. A total of 1 × 10^5^ cells were planted in each transwell chamber with or without matrix gel and incubated for 24 h or 48 h. The number of cells was counted after crystal violet staining.

### 4.6. Apoptosis Analysis

The apoptosis was detected using a commercial annexin V-FITC detection kit (Beyotime Biotechnology, Shanghai, China). After treatment with indicated concentrations of DHT, the cells were collected, washed twice with PBS, and resuspended with 100 μL of binding buffer. The cell suspension was incubated for 10 min at room temperature with 5 μL of annexin V-FITC and 10 μL of propidium iodide (PI). The percentage of apoptotic cells was detected by flow cytometry (Beckman CytoFlex, Brea 92822, CA, USA).

### 4.7. Extraction of PMNs and NETs

The PMNs were extracted from the femur bone of ICR mice according to the mouse bone marrow neutrophil separator kit (TBD, Tianjin, China). The PMNs were treated with indicated concentrations of PMA in RPMI-1640 medium. The supernatant was obtained by centrifugation at 250× *g* for 10 min. Then, the supernatant was centrifuged at 18,000× *g* for 20 min to obtain NETs and stored at −20 °C.

### 4.8. ROS Analysis

The PMNs were seeded in a 6-well plate (10^6^ cells/well), and treated with PMA (30 nM) and DHT (2 and 4 μM) for 8 h. Cells were collected at 1000 *g* for 5 min, then 1 μM of DCFH-DA solution (Solarbio, Beijing, China) was added to each well and incubated for 20 min. Thereafter, the cells were collected, washed twice with PBS, and detected at the FITC channel by flow cytometry.

### 4.9. Hoechst 33342 Staining

The PMNs were planted in a 24-well plate (10^5^ cells/well), and treated with PMA (15–60 nM) for 6 h. The cells were incubated with 1 μg/mL of hoechst 33342 solution for 30 min, washed with PBS for 3 times. The cell morphology was observed under a fluorescence microscope (Zeiss, Axio Scope. A1, Oberkochen 73450, BW, Germany).

### 4.10. dsDNA Test

The working solution was made up of dsDNA reagent: dsDNA buffer (1:199, *v*/*v*). The PMNs were treated with PMA and DHT; thereafter, 20 μL of the supernatant was collected and added into a 200 μL working solution. The solution was detected using the fluorescence microplate analyzer (Biotek, Synergy H1, Winooski 05404, VT, USA).

### 4.11. Western Blotting

The cells or tissues were lysed using RIPA buffer (Solarbio, Beijing, China) containing 1 mM of PMSF (Solarbio, Beijing, China) for 30 min on ice. The protein concentration was detected by the BCA protein assay kit (Yuanye Biotechnology, Shanghai, China). The protein samples (30 μg/lane) were subjected to SDS-PAGE (10–15%) and transferred to a PVDF membrane (Millipore, Massachusetts, USA). After blocking with 5% (*w*/*v*) non-fat milk for 2.5 h at room temperature, the membranes were incubated with primary antibodies at 4 °C overnight. After rinsing with TBST five times (5 min/time), the membranes were incubated with secondary antibodies, at room temperature, for 2.5 h. Finally, the bands were visualized using the ChemiSignal™ ECL Plus chemiluminescence solution (Clinx, Shanghai, China). The protein signal intensity was detected by imaging system (Tanon 4600SF, Shanghai, China) and quantified using the ImageJ software (National Institutes of Health, Bethesda 20816, MD, USA).

### 4.12. Animal Experiments

Female BALB/c nude mice (6-weeks old) were bought from the Shanghai Slack Laboratory Animal Company, Ltd. (SCXK (HU) 2017-0005). The mice were raised under pathogen-free conditions and allowed free access to sterilized food and water. The animal experiments were approved by the Animal Experimental Research Center of Zhejiang Chinese Medicine University, Zhejiang, China (SYXK (ZHE) 2021-0012). The 4T1 cells solved in RPMI-1640 medium (3 × 10^6^ cells/mice, n = 4) and matrigel were mixed and orthotopically injected into the mammary glands of the mice, in a ratio of 3:1 (*v*/*v*). The DHT was dissolved in a mixed solvent containing DMSO, PEG400 and saline (7:10:3, *v*/*v*/*v*). After five days, each mouse was intraperitoneally injected with DHT at 10 or 20 mg/kg/d marked as DHT-L and DHT-H, respectively. The control group was intraperitoneally injected with the same amount of solvent. The variations in body weight and tumor volume were recorded. After 25 days of inoculation, each mouse was anesthetized with isoflurane. Afterward, the mice were intraperitoneally injected with D-luciferin potassium salt (0.15 mg/g, solved in PBS). Finally, the fluorescence intensity of each mouse was measured by the FluoView400 Fluorescence Imaging system (BLT, Guangzhou, China) after 5 min of injection. The relative fluorescence intensity of the lung was calculated by the AniView software (BLT, Guangzhou, China).

### 4.13. Hematoxylin and Eosin Staining

Hematoxylin and eosin (H&E) staining was performed according to the standard procedures [40].

### 4.14. Immunofluorescence Analysis

The tissue slides were incubated with 0.1 M citrate buffer for 25 min, treated with 3% H_2_O_2_ for 20 min to quench endogenous peroxidase activity, permeabilized with 0.5% Triton X-100 for 30 min, and blocked with 1% bovine serum albumin in PBS for 30 min at room temperature. Thereafter, they were incubated with Mpo or Ly6G antibodies (1: 20) at 4℃ overnight. After rinsing with PBS, they were incubated with goat anti-rabbit immunoglobulin G and Alexa Fluor 488 (Invitrogen, USA) at room temperature for 1 h in darkness. ProLong Gold Antifade Mountant containing DAPI (Invitrogen, Carlsbad 92008, CA, USA) was used to stain the nucleus. Fluorescence intensity was calculated by the Image Pro Plus 6.0 software (Media Cybernetics, Silver Spring 20910, MD, USA).

### 4.15. Immunohistochemistry

The tissue slides were incubated with 0.1 M citrate buffer for half an hour and then treated with 3% hydrogen peroxide for 18 min to quench endogenous peroxidase activity. The slides were blocked with 10% goat serum for 1 h at room temperature, then primary antibody Ki67 was added and stained with 3,3′-diaminobenzidine. Nuclei were stained with hematoxylin. Images were captured under a microscope (Zeiss AXIO SCOPE A1, Germany). The Image Pro Plus software was used to analyze the results.

### 4.16. Molecular Docking

The structures of *TIMP1* (7S7M) and *SerpinB2* (1BY7) were downloaded from the PDB database (https://www.rcsb.org/, accessed on 7 July 2022). The structure of DHT was downloaded from the PubChem database (https://pubchem.ncbi.nlm.nih.gov/, accessed on 7 July 2022). The molecular docking process is completed by AutoDockTools-1.5.7. The display diagram of molecular docking results is modified with Pymol.

### 4.17. TIMER Database Analysis

TIMER (https://cistrome.shinyapps.io/timer/, accessed on 21 February 2022) is a comprehensive database for analyzing immune infiltration in different cancer types [41]. Diff Exp module was selected and *HIST3H3* (the gene of H3cit) was input to obtain the correlation between *HIST3H3* and different cancers. The parameters were as follows: module, Gene; gene symbol, *HIST3H3*; cancer types, BRCA; immune infiltration, and neutrophils. The correlation diagram between *HIST3H3* and neutrophil infiltration in BC was analyzed. The SCNA module, *HIST3H3*, BRCA, and neutrophil were selected to compare the different stages of *HIST3H3* and neutrophil infiltration in BC.

### 4.18. RNA Sequencing

The PMNs were treated with DHT (2 μM and 4 μM) and PMA (30 nM) for 8 h. All PMNs were collected and washed with PBS twice. The RNA of each group was extracted, and the purity was determined using NanoDrop (Invitrogen, USA). The qualified RNA was selected for amplification and establishment of cDNA library. The library was sequenced using the Illumina HiSeq™ 300 platform. The original sequence data was filtered using HISAT. The clean reads of each sample were sequenced with the specified reference genome. The gene expression was quantified by FPKM value with *p* < 0.05 and |log2 (fold change)| ≥ 2 as a confidence threshold.

### 4.19. Bioinformatics Analysis

The differential genes were conducted by GO (Gene Ontology, http://www.geneontology.org/, accessed on 16 May 2022), KEGG (Kyoto Encyclopedia of Genes and Genomes, http://www.genome.jp/kegg/, accessed on 16 May 2022), Reactome (https://reactome.org/, accessed on 16 May 2022), and bioinformatics (http://www.bioinformatics.com.cn/, accessed on 16 May 2022). The protein-protein interaction network of different genes was analyzed using the STRING database (https://cn.string-db.org/, accessed on 16 May 2022) with a confidence threshold of 0.2 or 0.4. The Human Protein Atlas database (https://www.proteinatlas.org/, accessed on 16 May 2022) is an open database related to human proteins and genes [42]. It was used to analyze the protein expression in patients. *TIMP1* was input into the search box, respectively, and then select the immunohistochemical images of the breast in normal population and breast cancer patients.

### 4.20. Statistical Analysis

All of the results were represented as mean ± standard deviation (SD). The differences between groups were analyzed by a student′s *t*-test or one-way ANOVA, supported by IBM SPSS Statistics 26.0 software (SPSS Inc., Chicago 60606, IL, USA). *p* < 0.05 was considered as statistically significant.

## 5. Conclusions

This study demonstrated that DHT suppressed the neutrophil infiltration and NETs formation, which subsequently inhibited lung metastasis, of BC. The effect of NETs on the proliferation and migration of the 4T1 BC cells could be reversed by DHT. In addition, the anti-lung metastasis mechanism of DHT might be related to *TIMP1*-mediated NETs inhibition (Figure 7D). DHT may become a novel NETs inhibitor for the treatment of BC lung metastases by targeting *TIMP1*.

## Data Availability

The data presented in this study are available on request from the corresponding author. The data are not publicly available due to continued deeper research.

## Supplementary Materials

The following supporting information can be downloaded at: https://www.mdpi.com/article/10.3390/ijms232315180/s1, Figure S1: The effects of DHT, DDP, and PTX on the proliferation of normal breast epithelial cells MCF-10a; Figure S2: The effects of PTX and DDP on clonogenicity of BC cells; Figure S3: The effects of DHT on PMNs morphology; Table S1: The IC_50_ (μM) of four tanshinones, DDP, and PTX on BC cells and normal breast epithelial cells; Table S2: List of differentially expressed genes from the control vs. PMA (up group) and the PMA vs. P + DHT (down group) (|log2fold change| ≥ 2); Table S3: List of PPI results.
