# Supplementary material for: Dihydrotanshinone I Inhibits the Lung Metastasis of Breast Cancer by Suppressing Neutrophil Extracellular Traps Formation
Source: Int J Mol Sci. 2022 Dec 2;23(23):15180. doi: 10.3390/ijms232315180 (PMC9736467; doi:10.3390/ijms232315180)
Supplement: Supplementary file 1 [file ijms-23-15180-s001.zip › ijms-2040222-supplementary.pdf]

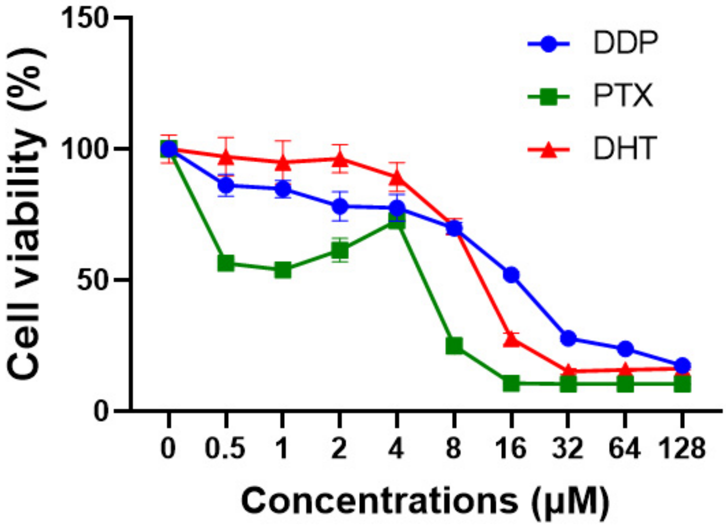


**Figure S1**. The effects of DHT, DDP, and PTX on the proliferation of normal breast epithelial cells MCF-10a. MCF-10a cells were treated with DHT or positive drugs DDP and PTX (1-128 μM) for 24 h, respectively. The $IC_{50}$ for MCF-10a is 13.58 μM (DHT), 14.46 μM (DDP) and 2.06 μM (PTX). Data were represented as mean ± SD, n = 3.

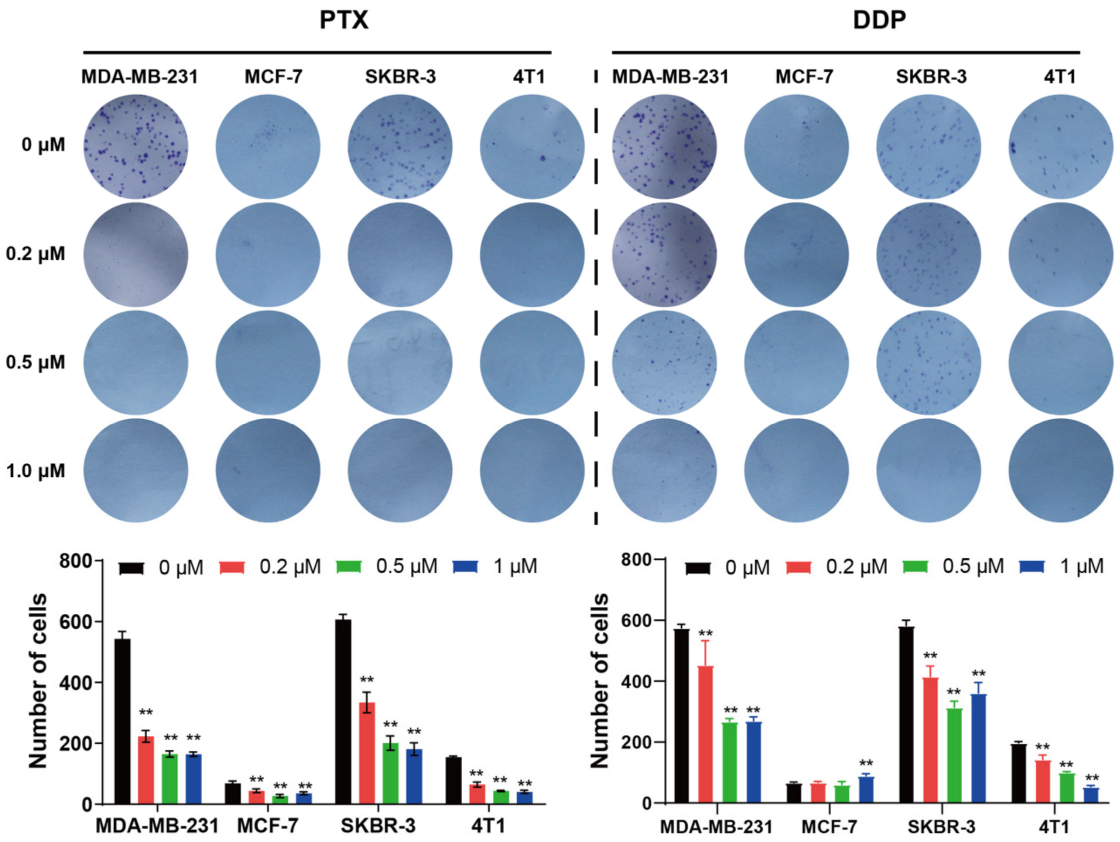


**Figure S2**. The effects of PTX and DDP on clonogenicity of BC cells. MDA-MB-231, MCF-7, SKBR-3, and 4T1 cells were treated with PTX or DDP (0.2-1.0 μM) for 8 d. Clonogenicity was determined by crystal violet staining. Data were represented as mean ± SD, n = 3. **p* < 0.05 and ***p* < 0.01 compared with the control group.

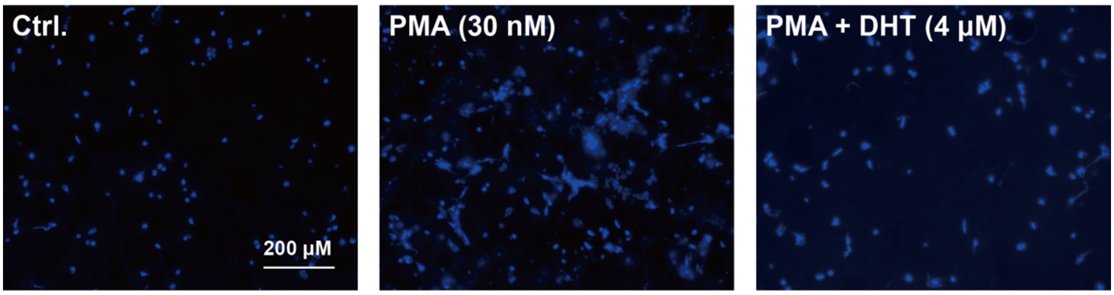


**Figure S3**. The effects of DHT on PMNs morphology. PMNs were treated with PMA (30 nM) or PMA (30 nM) + DHT (4 μM) for 6 h. The cell morphology was determined by Hoechst 33342 staining.

**Table S1**. The $IC_{50}$ (μM) of four tanshinones, DDP, and PTX on BC cells and normal breast epithelial cells.

| Compounds | MDA-MB-231 | MCF-7 | SKBR-3 | 4T1 | MCF-10a |
|---|---|---|---|---|---|
| DHT | 117.71 | 34.11 | 17.87 | 6.97 | 13.58 |
| Tan I | 295.89 | 106.33 | 131.71 | 152.22 | N.D. |
| Tan IIA | 571.30 | 448.00 | 1120.75 | 134.74 | N.D. |
| CPT | 544.01 | 128.69 | 269.08 | 27.91 | N.D. |
| DDP | 2613.12 | 50.90 | 134.93 | 51.53 | 14.46 |
| PTX | 30.85 | 8.17 | 9.62 | 5.08 | 2.06 |

Note: N.D. means not detection.
